# Regulation of T Cell Motility *In Vitro* and *In Vivo* by LPA and LPA2

**DOI:** 10.1371/journal.pone.0101655

**Published:** 2014-07-08

**Authors:** Sara A. Knowlden, Tara Capece, Milan Popovic, Timothy J. Chapman, Fariba Rezaee, Minsoo Kim, Steve N. Georas

**Affiliations:** 1 Department of Microbiology and Immunology, University of Rochester Medical Center, Rochester, New York, United States of America; 2 Division of Pulmonary and Critical Care Medicine, Department of Medicine, University of Rochester Medical Center, Rochester, New York, United States of America; 3 Division of Pediatric Pulmonary Medicine, Department of Pediatrics, University of Rochester Medical Center, Rochester, New York, United States of America; University of Pécs Medical School, Hungary

## Abstract

Lysophosphatidic acid (LPA) and the LPA-generating enzyme autotaxin (ATX) have been implicated in lymphocyte trafficking and the regulation of lymphocyte entry into lymph nodes. High local concentrations of LPA are thought to be present in lymph node high endothelial venules, suggesting a direct influence of LPA on cell migration. However, little is known about the mechanism of action of LPA, and more work is needed to define the expression and function of the six known G protein-coupled receptors (LPA 1–6) in T cells. We studied the effects of 18∶1 and 16∶0 LPA on naïve CD4+ T cell migration and show that LPA induces CD4+ T cell chemorepulsion in a Transwell system, and also improves the quality of non-directed migration on ICAM-1 and CCL21 coated plates. Using intravital two-photon microscopy, *lpa2−/−* CD4+ T cells display a striking defect in early migratory behavior at HEVs and in lymph nodes. However, later homeostatic recirculation and LPA-directed migration *in vitro* were unaffected by loss of *lpa2*. Taken together, these data highlight a previously unsuspected and non-redundant role for LPA2 in intranodal T cell motility, and suggest that specific functions of LPA may be manipulated by targeting T cell LPA receptors.

## Introduction

Lymphocyte trafficking to immune organs and peripheral tissues is a highly regulated process that has emerged as a critical checkpoint in the initiation and outcome of immune responses. A current paradigm in immunology is that different lymphocyte subsets exhibit different homing properties *in vivo* that are regulated by chemokines, adhesion molecules, and lipid mediators. Recently, the enzyme autotaxin (ATX) has been shown to be constitutively expressed at the high endothelial venules (HEV) of lymph nodes and potentially regulate lymphocyte entry. ATX possess integrin binding motifs that allow it to bind to the leading edge of migrating human T cells in a β1 integrin-dependent manner, suggesting it may play a role in lymphocyte arrest and/or transendothelial migration [1]–[3]. ATX expression is independent of HEV-associated chemokines or MyD88-dependent signals, highlighting a potential unique function for ATX in the T cell homing process [3].

A major enzymatic role for ATX is its lysophospholipase D activity, whereby ATX cleaves the choline group from lysophosphatidylcholine (LPC) to generate lysophosphatidic acid (LPA) [4]. LPA is a pluripotent extracellular lysolipid that has physiological roles in the cardiovascular system as a mediator of angiogenesis [5]–[8], vascular maturation [9], [10], and wound repair [11], as well as pathologic roles in disease states (reviewed in [12]) such as atherosclerosis [13]–[15], cancer [16]–[20], lung fibrosis [21]–[25], arthritis [26]–[29] and asthma [30]–[32]. Emerging data also point to important roles for LPA in the immune system including lymphocyte trafficking [2], [3], [33]–[35].

Interestingly, Kanda et al. showed that LPA induces human T cell chemokinetic activity (not chemotaxis) [2], while Zhang et al. demonstrated that LPA stimulated uropod formation and polarization of T cells *in vitro* [33]. When T cells were pre-treated with HA130, a mutant form of ATX, and adoptively transferred into mice, the acute migration of T cells across HEVs was attenuated [2]. These studies suggest that constitutive ATX expression by HEVs generates high LPA levels in the vicinity of the HEV due to catalysis of endogenous LPC, and may promote lymphocyte egress from the blood stream into lymph nodes in an LPA-dependent manner. However, the role of individual LPA receptors in promoting T cell migration has not yet been explored.

LPA elicits its effects by binding to at least six known G protein-coupled receptors (LPA1–6). These receptors are divided into two subgroups based on their primary sequences; LPA1–3 belong to the endothelial differentiation gene (Edg) family of receptors [36]–[38], while LPA4–6 belong to the purinergic P2Y receptor family [39]–[41]. The LPA receptors are expressed in a wide variety of tissues and cells, including high mRNA expression of LPA1 in the brain [42], LPA2 in a number of cancers [43]–[46], LPA3 in the uterus [47], [48], LPA1 and LPA4 on MAdCAM-1+ endothelial cells [3], LPA5 in the small intestine on CD8+ intraepithelial lymphocytes [49], and LPA6 in hair follicles [50]. Signaling through LPA receptors can affect the proliferation, growth, activation, and migration of many different cell types, and each receptor couples to at least two downstream G-protein pathways. Studying surface LPA receptor expression is technically challenging due to a lack of antibodies that detect LPA receptors by flow cytometry, and how different LPA receptors influence T cell migration is poorly understood.

We studied the expression and function of individual T cell LPA receptors in naïve and activated CD4+ cells using *in vitro* migration assays and adoptive transfer strategies. We compared cells from wild-type and LPA2 gene-targeted mice, and studied both directed and non-directed migration *in vitro*, as well as both early and late accumulation of T cells in lymph nodes after adoptive transfer. Here we report that mouse CD4+ T cells differentially express six LPA receptors and that expression of these receptors changes over the course of T cell activation and polarization. The absence of LPA2 does not change the chemokinetic effect of LPA in a Transwell system or homeostatic naïve CD4+ T cell re-circulation after adoptive transfer. However, LPA2 KO CD4+ T cells displayed a striking defect in the early homing process at HEVs, as seen using two-photon intravital microscopy.

## Materials and Methods

### Mice

Wild-type C57BL/6 and C57BL/6.PL (CD90.1, CD45.2) mice were purchased from The Jackson Laboratory. LPA2-deficient (*lpa2*−/−) mice were derived from frozen gene-targeted embryos provided by Deltagen (San Mateo, CA), in collaboration with GlaxoSmithKline (GSK) and backcrossed for more than six generations onto the C57BL/6 background. C57BL/6.SJL (CD90.2, CD45.1) OT-II TCR transgenic mice recognizing OVA peptide OVA_323–339_ in the context of I^ab^ were a gift from Dr. Troy Randall (University of Rochester). All mice were maintained at the University of Rochester and age- and gender-matched littermate controls were used in all experiments. The studies were carried out in strict accordance with the recommendations in the Guide for the Care and Use of Laboratory Animals of the National Institutes of Health. The protocol was reviewed and approved by the University of Rochester Committee of Animal Resources and studies were conducted in accordance with institutional guidelines.

### Antibodies and reagents

The antibodies used were purified anti-mouse CD3e and CD28, PE-conjugated anti-CD3e, APC-conjugated anti-CD4, Pacific Blue-conjugated anti-CD44 from BioLegend (San Diego, CA); anti-IL-4 (clone 11B11) and anti-IFN-γ (clone XMG1.2) from BioXCell (West Lebanon, NH); FITC-conjugated anti-CD90.2 from BD Biosciences (San Jose, CA); APC-AF780-conjugated anti-CD45.1 from eBioscience (San Diego, CA). Western blot antibodies LPA1 (3 µg/mL; ab23698), LPA2 (1∶250; ab38322), and LPA3 (1∶200; ab23692) were from Abcam (Cambridge, MA). The cytokines and chemokines used were recombinant human IL-2 from BD Biosciences; mouse IL-6, IL-4, and TGF-β from Peprotech (Rocky Hill, NJ); mouse IL-23 and CCL21, and recombinant mouse ICAM-1 from R&D Systems, Inc. (Minneapolis, MN). Recombinant Protein A and propidium iodide were from Invitrogen (Carlsbad, CA).

### Preparation of LPA

All LPA stocks (18∶1 and 16∶0; Avanti Polar Lipids, Alabaster, AL) were purchased in powder form and dissolved in chloroform+1.2% methanol+0.6% water and stored at −80°C. Some lots of 18∶1 LPA were purchased dissolved in chloroform. Fresh LPA was used for each experiment and was first dried down with nitrogen gas, resuspended in PBS+1% fatty-acid free BSA (Sigma-Aldrich, St. Louis, MO), and vortexed and sonicated until LPA was in solution. We noted lot-to-lot variability in the ability of 18∶1 LPA to dissolve in aqueous solution, and only used LPA solutions that showed clear solubility as determined by visual inspection.

### Mouse CD4+ T cells

Peripheral lymph nodes and spleens were harvested from mice and processed to single cell suspensions by mechanical homogenization. Red blood cells were lysed using 1x RBC Lysis Buffer (eBioscience, San Diego, CA). CD4+ T cells were enriched by negative selection using a MACS CD4+ T cell Isolation Kit (Miltenyi Biotec, Auburn, CA). To activate T cells, the cells were cultured with plate-bound antibodies against CD-3 (500 ng/ml) and CD-28 (2 µg/ml) for 24, 48, or 72 hours in the presence of recombinant human IL-2. To polarize CD4+ T cells, cells were cultured with plate-bound antibodies against CD3 and CD28 and the following conditions- Th1: IL-12, anti-IL-4, IL-2; Th2: IL-4, anti-IFN-γ, IL-2; Th17: TGF-β-1, IL-6, anti-IL-4, anti-IFN-γ.

### Chemotaxis assay

Mouse CD4+ T cells (2.5×10^5^) in serum-free media were added to the top chambers of Transwells (pore size 5 µm; Corning, Lowell, MA). LPA (1 µM 18∶1 and 16∶0; Avanti Polar Lipids) was diluted in serum-free media and was added to either the top or bottom chamber or both chambers and the cells were allowed to migrate for 2 hours at 37°C. As a positive control for chemotaxis, CCL21 (100 ng/ml) was added to the bottom chambers. The number of migrated cells was quantified by hemocytometry.

### Non-directed migration

Delta T dishes were coated with Protein A (20 µg/ml in PBS) overnight at 4°C. The dishes were then incubated at room temperature for 4 hours before being washed with PBS and then incubated with ICAM-1 (10 µg/ml) and CCL21 (200 ng/ml) for 2 hours. Purified CD4+ T cells were resuspended in Leibovitz’s L15 media with no phenol red with or without LPA (1 or 10 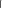
M; 18∶1 and 16∶0) and allowed to settle for 15 minutes before imaging. Image acquisition was conducted on an epifluorescence microscope (TE2000-U microscope; Nikon) using 20x objectives coupled to a Cool-SNAP HQ CCD (Roper Scientific). Differential interference contrast (DIC) images were acquired every 5 s for 15 min and 37°C was maintained throughout the experiment. Volocity software was used for image analysis and tracking (Improvision).

### Assay of LPA receptor expression by real-time PCR

At 0, 24, 48, or 72 hours post-activation with anti-CD3 and anti-CD28 antibodies, cells were harvested and resuspended in TRIzol (Life Technologies, Grand Island, NY) for RNA extraction. cDNA synthesis was performed using iScript cDNA Synthesis Kit (Bio-Rad Life Science, Hercules, CA) according to the manufacturer’s instructions. Semi-quantitative real-time PCR was performed using the iQ SYBR Green Supermix Assay System (Bio-Rad) and PCR amplifications were performed in triplicate on the iQ5 Multicolor real-time PCR detection system (Bio-Rad). The following forward and reverse primers were synthesized by Integrated DNA Technologies: LPA1, forward 5′-CTATGTTCGCCAGAGGACTATG-3′ and reverse 5′-GCAATAACAAGACCAATCCCG-3′; LPA2, forward 5′-CACACTCAGCCTAGTCAAGAC-3′ and reverse 5′-GTACTTCTCCACAGCCAGAAC-3′; LPA3, forward 5′-ACCAACGTCTTATCTCCACAC-3′ and reverse 5′-CAGTTCAGGCCGTCCAG-3′; LPA4, forward 5′-AGGATGGAGTCGCTGTTTAAG-3′ and reverse 3′-CTAACTTCCTCTTGGATAGCTGG-3′; LPA5, forward 5′-TGGAGGTGAAAGTCATGCTC-3′ and reverse 5′-GTATCTCGATAGRCAGGGCAC-3′; LPA6, forward 5′-CACATCTGAATAGCAAAGGCG-3′ and reverse 5′-TGAACATGCACCCGTACAG-3′. Quantification of LPA receptor expression was performed by the 2∧-deltaCt method (Relative Value Units (RVU) = 2∧-deltaCt×1000) or the 2∧-deltadeltaCt method. The normalized Ct values were calculated using mouse GAPDH mRNA expression as an endogenous control.

### Western blot for LPA1, LPA2, LPA3

At 0, 24, and 72 hours post-activation with anti-CD3 and anti-CD28 antibodies, CD4+ T cells were harvested, washed two times with cold PBS, and lysed in RIPA lysis buffer supplemented with protease inhibitors (Sigma). The protein concentrations were quantified by bicinchoninic acid protein assay (Pierce, Cheshire, United Kingdom), followed by resolution on 10% SDS-PAGE gel and transferred to polyvinylidene difluoride (PVDF) membranes (Bio-Rad). The membranes were blocked with 5% milk/TBST (for LPA1, LPA2) or 0.5% milk/TBST (for LPA3) for one hour at room temperature, followed by overnight incubation with indicated primary antibodies at 4°C, and then for 1 hour at room temperature with horseradish peroxidase-conjugated secondary antibodies. The blots were exposed to enhanced chemiluminescence (ECL; Bio-Rad) and subjected to autoradiography. Glyceraldehyde-3-phosphate dehydrogenase (GAPDH; Abcam) was used as a lane loading control.

### Multi-photon intravital imaging

Wild-type CD4+ T cells from C57BL/6 mice were labeled for 5 min at 37°C with 1.25 µM 5-(and 6)-carboxyfluorescein diacetate succinimidyl ester (CFSE; Life Technologies, Invitrogen). *Lpa2−/−* CD4+ T cells were labeled for 20 min at 37°C with 10 µM 5-(and-6)-(((4-chloromethyl)benzoyl)amino)tetramethylrhodamine (CMTMR; Life Technologies, Invitrogen). Blood vessels were visualized by Texas Red Dextran (20 mg/kg body weight, 70 kDa molecular weight, Life Technologies, Invitrogen). 5–10×10^6^ cells mixed at 1∶1 ratio together with Texas Red Dextran were given to WT recipients by injection into orbital sinus just before starting to image. Mice were anesthetized by an initial intraperitoneal injection of sodium pentobarbital, at a dose of 65 mg/kg body weight and placed in the custom-made chamber with pre-warmed normal saline. The right popliteal lymph node was exposed microsurgically and additional precaution was taken to spare blood vessels and afferent lymph vessels. The core body temperature of the mouse was maintained using a warming plate set to 37°C. To avoid psychological stress and pain of the animal during imaging, further anesthesia was maintained using isoflurane. To visualize T cell motility during extravasation, MP-IVM was performed using an FV1000-AOM multiphoton system (Olympus) equipped with a 25×NA1.05 water immersion objective. For two-photon excitation, a MaiTai HP Ti:Sa Deep See laser system (Spectra-Physics) was tuned to 840 nm for CFSE/CMTMR and 900 nm for CFSE/Texas red. The images were acquired at a resolution of 256×256 pixels, with a pixel dwell time of 2 µs, using step sizes of 2 µm to a depth of 50 µm every 45 s. CFSE, and CMTMR/Texas red were visualized using band-pass filters with 495/560 nm and 575/630 nm, respectively. Raw imaging data were processed using Volocity software (Perkin Elmer). A median filter was used to control background noise and T cell tracks were determined using automated algorithms with manual corrections. Cell tracks lasting less than 5 minutes were excluded from analyses and no minimum displacement criteria was applied in order not to exclude nonmotile cells.

### *Lpa2−/−* competitive adoptive transfer

CD4+ T cells were enriched from the lymph nodes and spleens of wild-type C57BL/6.SJL OT-II TCR transgenic mice and *lpa2−/−* OT-II TCR transgenic mice. An equal number of WT and *lpa2−/−* CD4+ T cells (1.5×10^6^ of each) were adoptively transferred through tail vein injection into wild-type C57BL/6.PL mice. Forty-two hours after adoptive transfer, inguinal, brachial, cervical lymph nodes, and spleen were harvested. The number of donor CD4+ T cells was enumerated by flow cytometry. Live wild-type cells were gated on CD3+CD4+CD44lowCD90.2+CD45.1+ and live *lpa2−/−* cells were gated on CD3+CD4+CD44lowCD90.2+CD45.1−.

### Statistical analysis

Statistical analysis was performed using GraphPad Prism 6 software. The unpaired Student t test was used to determine the statistical significance of pair-wise comparisons. For comparisons of three or more groups, one-way ANOVA with the Tukey’s multiple comparisons post-test was used.

## Results

### LPA induces chemorepulsion of naïve CD4+ T cells

**Figure 1:**
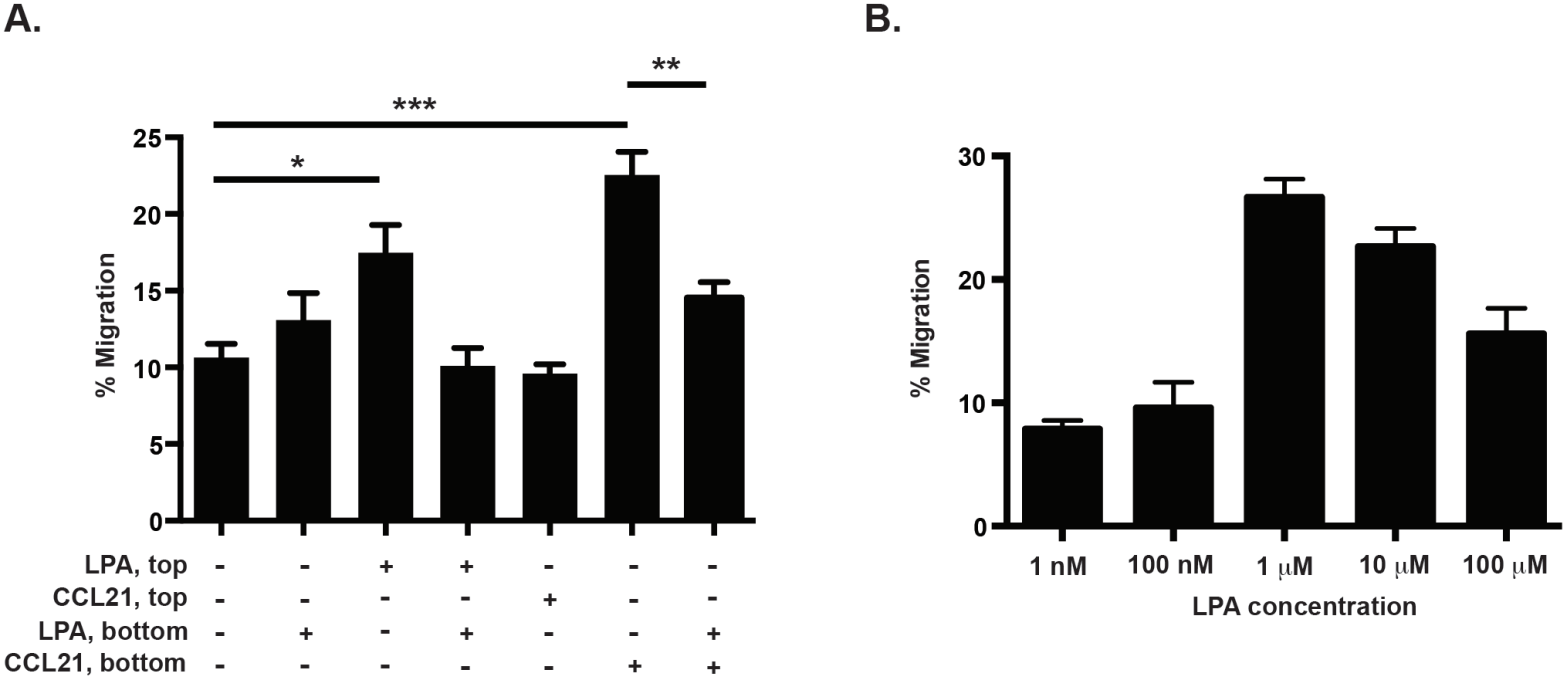
LPA induces the migration, but not chemotaxis of naïve CD4+ T cells. (**A.**) Naïve mouse CD4+ T cells were added to the upper chamber of a Transwell and LPA (1∶1 mixture of 18∶1 and 16∶0 LPA at 1 µM final concentration) was added to the bottom chamber or top chamber as indicated or (**B.**) top chamber at various concentrations. Cells were allowed to migrate for 2 hours at 37°C and the number of cells that migrated to the bottom chamber was quantified by hemocytometry. CCL21 (100 ng/ml) was added to the bottom chamber to induce chemotaxis, as a positive control. Data are mean +/− SEM of 3–5 experiments. *p<0.05; **p<0.01; ****p<0.0001.

In order to gain a better understanding of how LPA affects directed cell migration, we first used standard chemotaxis Transwell assays. Wild-type naïve CD4+ T cells were harvested from mice and a known number of cells were added to the top chamber of a Transwell. Serum-free media containing LPA and/or CCL21 were added to either the top or bottom chambers, as shown in **Figure 1A**. When LPA was added to the bottom chamber, there was no increase in migration of naïve CD4+ T cells to the bottom chamber, indicating that LPA does not induce chemotaxis (**Figure 1A**, second bar). However, when LPA was added to the top chamber, we recovered significantly more cells from the bottom chamber, but we did not detect a significant increase in migration of the cells above baseline when LPA was added to both the top and bottom chambers. We next determined whether LPA interacted with CCL21, a chemokine involved in naïve T cell homing to lymph nodes. As expected, addition of CCL21 to the bottom chamber enhanced T cells chemotaxis (**Figure 1A**, second to last bar). Interestingly, co-incubation of LPA and CCL21 in the bottom chamber attenuated T cell migration towards CCL21 (**Figure 1A**, last bar). These data indicate that LPA does not act as a standard chemoattractant in vitro (in keeping with prior studies [2]), but instead appears to induce naïve CD4+ T cell chemorepulsion. These findings prompted us to perform a dose response experiment with increasing concentrations of LPA in the top chamber (1 nM-100 µM; **Figure 1B**). We saw a bell-shaped response with increasing concentrations of LPA, with 1 µM LPA eliciting the greatest chemorepulsion. We used 1 µM LPA in subsequent in vitro experiments.

### LPA enhances non-directed migration of naïve CD4+ T cells

**Figure 2:**
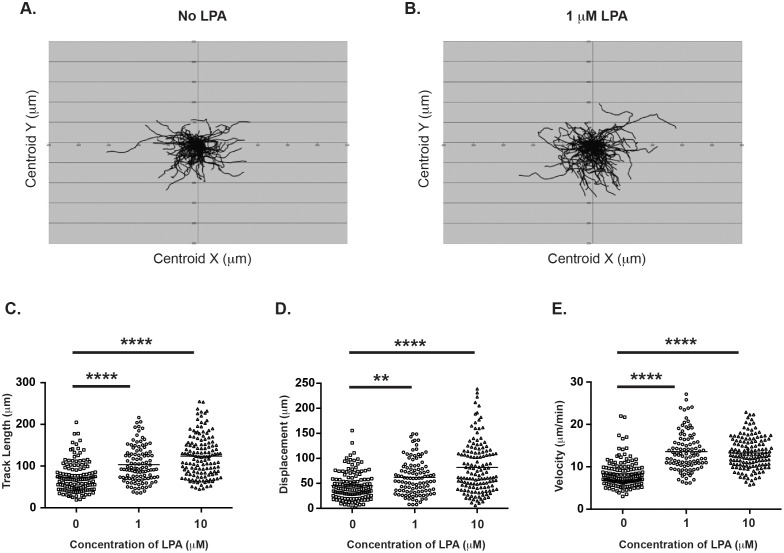
LPA induces enhanced migration of naïve CD4+ T cells. Naïve mouse CD4+ T cells from wild-type C57BL/6 mice were added to microchambers coated with ICAM-1 and CCL21 in the absence of LPA or with 1 µM or 10 µM LPA. T cell migration was imaged every 15 s for 15 min and tracked by Volocity software. (**A**). Spider-plots of individual cell tracks over 15 min without LPA or with 1 µM LPA. (**B**). Mean track length, (**C**). Mean displacement, (**D**). Mean velocity with (1 µM and 10 µM) LPA or without LPA. Data are mean +/− SEM and representative of three independent experiments. **p<0.01, ****p<0.0001.

We next studied the effects of LPA on non-directional migration by examining T cell movement on ICAM-1 and CCL21 coated plates in the presence or absence of LPA. As seen in **Figure 2A**, in the absence of LPA, naïve CD4+ T cells migrate in a typical random fashion, with a mean track length of 73.44 µm (**Figure 2C**), displacement of 45.42 µm (**Figure 2D**), and a mean velocity of 8.32 µm/min (**Figure 2E**). However, when 1 µM or 10 µM of LPA was added (**Figure 2B**), the migration pattern changed. Specifically, cells migrated longer distances, as seen by increased track length of 103.4 µm and 123.8 µm (p<0.0001) (**Figure 2C**), farther from their point of origin, with increased displacement from 62.03 µm to 81.96 µm (p<0.01 and p<0.0001, respectively) (**Figure 2D**), and at a higher mean velocity of 13.56 µm/min and 13.27 µm/min (<0.0001) (**Figure 2E**), respectively. Taken together, these data reveal that LPA enhances the quality of migration of naïve CD4+ T cells on an ICAM-1 and CCL21-coated surface. Our results corroborate the findings of Zhang et al. who showed that either LPA or ATX plus LPC induce non-directed T cell motility in vitro [33].

### CD4+ T cells have distinct LPA receptor profiles

**Figure 3:**
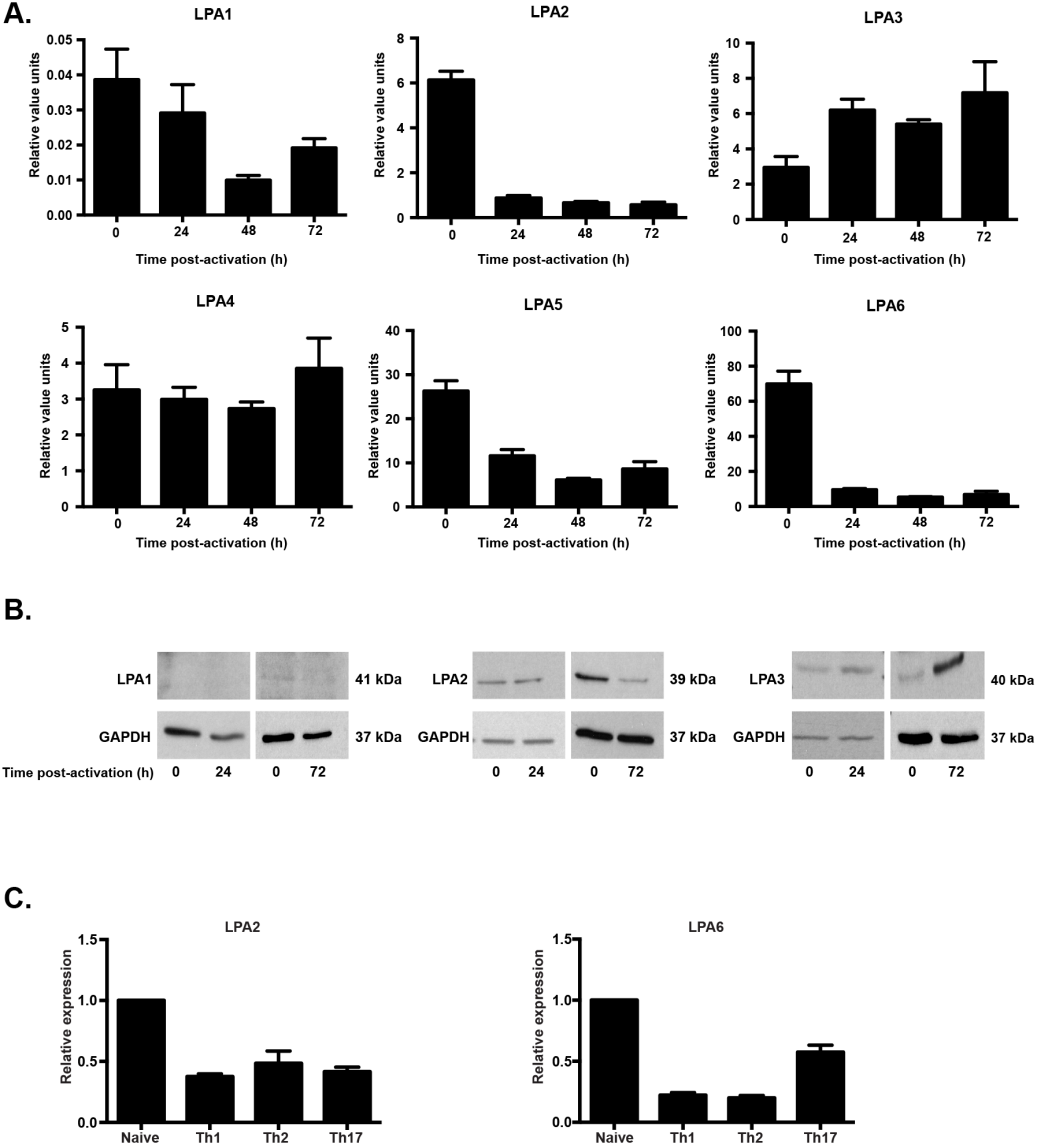
Mouse CD4+ T cells differentially express LPA1–6 over the course of T cell activation and polarization. (**A**). Naïve mouse CD4+ T cells were collected and activated by plate-bound anti-CD3 and anti-CD28 antibodies for 24, 48, or 72 hours. At each time point, cells were harvested and the mRNA expression of LPA1,2,3,4,5,6 was determined by semi-quantitative real-time PCR, performed in triplicate. Expression levels were normalized to mouse GAPDH using the 2∧-deltaCt method. Relative Value Units (RVU) = 2∧-deltaCt×1000. Data are mean of three independent experiments. (**B**). Protein lysates were collected at 0, 24, and 72 hours post-activation and protein expression of LPA1, LPA2, and LPA3 was measured by Western blot. GAPDH was used as a lane loading control. Data are representative of one-two experiments. (**C**). Naïve mouse CD4+ T cells were collected and activated by plate-bound anti-CD3 and anti-CD28 antibodies and cultured under Th1 (IFN-γ, anti-IL-4), Th2 (IL-4, anti-IFN-γ), or Th17 (TGF-β, IL-6, anti-IL-4, anti-IFN-γ) polarizing conditions for 72 hours. Cells were harvested and the mRNA expression of LPA2 and LPA6 was determined by semi-quantitative real-time PCR, performed in triplicate. Expression levels were normalized to mouse GAPDH and compared to naïve CD4+ T cells using the 2∧-deltadeltaCt method. Data are mean +/− SEM of three independent experiments.

Very little is known about the expression of LPA receptors in resting and activated T cells. Surface expression of LPA receptors is difficult to study due to the lack of antibodies that reliably detect the extracellular domains and work in flow cytometry (to our knowledge). To this end, we used real-time PCR to examine LPA receptor mRNA expression in resting naïve CD4+ T cells, as well as over the course of T cell activation. Mouse CD4+ T cells were stimulated with anti-CD3 and anti-CD28 antibodies and cells were harvested at 0, 24, 48, and 72 hours post-activation. Interestingly, expression of the six LPA receptors was differentially regulated over the course of activation (**Figure 3A**). LPA2, 3, 5, and 6 were most highly expressed in naïve CD4+ T cells (note different scales in Figure 3), and expression of LPA2, 5, and 6 decreased after activation. However, LPA3 expression increased upon activation. Expression of LPA1 was near the detection limit and both LPA1 and LPA4 mRNA expression did not change after cell stimulation. We confirmed these results at the protein level by examining expression of LPA1, LPA2, and LPA3 by Western blot in resting and activated CD4+ T cells (**Figure 3B**). LPA1 was undetectable, while protein expression of LPA2 decreased after 72 hours of activation. Similarly to the mRNA, the protein expression of LPA3 increased after activation, highlighting the possibility that LPA3 functions in activated T cells.

We studied the expression and function of LPA2 in more detail, since LPA2 couples to both Gαi and Gα12/13, which have been implicated in LPA-directed T cell migration and uropod formation [2], [33]. Although we determined that LPA2 expression decreases after anti-CD3+CD28-dependent T cell activation, we next studied LPA2 mRNA under Th1 (IFN-γ, anti-IL-4), Th2 (IL-4, anti-IFN-γ), and Th17 (IL-6, TGF-β, anti-IL-4, anti-IFN-γ) polarizing conditions. For comparison, we measured expression of LPA6, which also couples to both Gαi and Gα12/13 but has not been well-studied in lymphocytes. Similarly to when CD4+ T cells were acutely activated in vitro, the expression of both LPA2 and LPA6 decreased when CD4+ T cells were differentiated into Th1, Th2, and Th17 lineages (**Figure 3C**). In subsequent experiments, we studied naïve and unpolarized T cells.

### The absence of lpa2 does not affect LPA-induced chemorepulsion

**Figure 4:**
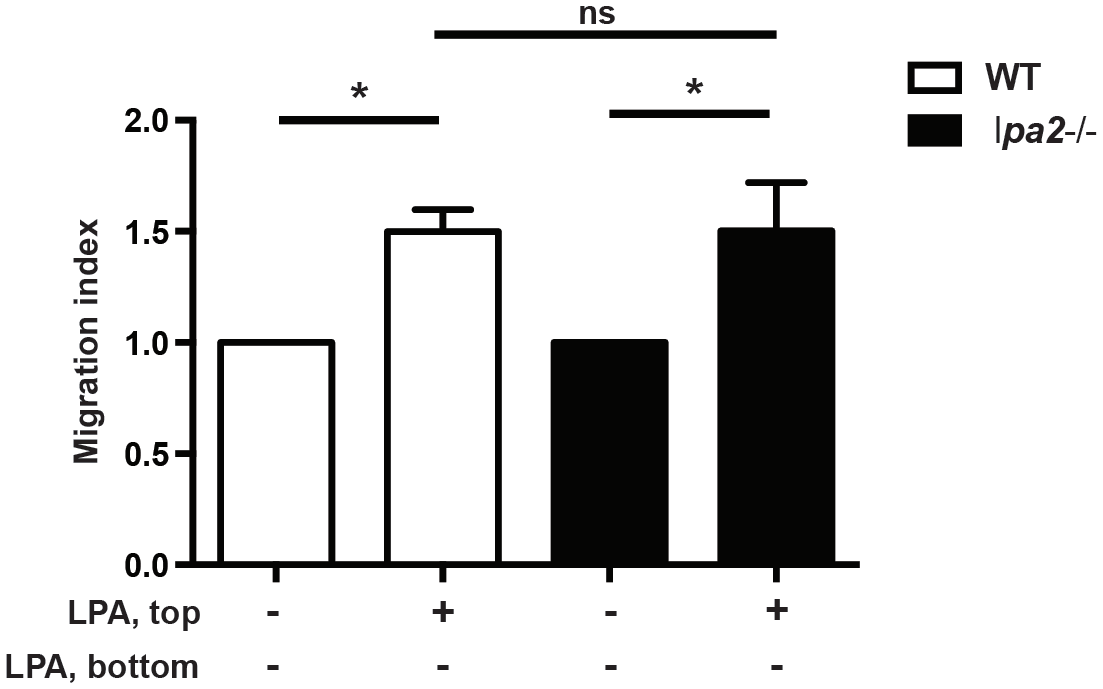
Naïve CD4+ T cells from *lpa2*−/− mice still migrate in response to LPA. Naive mouse CD4+ T cells from wild-type C57BL/6 mice (open bars) or *lpa2*−/− mice (dark bars) were added to the upper chamber of a Transwell (5 µm pore size) in the presence or absence of LPA (1 µM; 18∶1 and 16∶0). Cells were allowed to migrate for 2 hours at 37°C and cell migration analyzed as in Figure 2. Data are expressed as Migration Index, or the number of cells that migrated in response to LPA relative to the number of cells that migrated in the presence of serum-free media only. Data are mean +/− SEM of four independent experiments. *p<0.05. n.s. = not significant.

To investigate the role of LPA2 in promoting LPA-induced migration, we compared naïve CD4+ T cells from *lpa2*−/− mice in parallel Transwell assays. In **Figure 4**, wild-type or *lpa2*−/− naïve CD4+ T cells were added to the top chamber in the presence of 1 µM LPA and allowed to migrate. After 2 hours there was no difference in LPA-induced migration, indicating that both wild-type and *lpa2−/−* CD4+ T cells migrated away from LPA. Thus in the Transwell assay at least, LPA2 is not required for directional migration induced by LPA. In later experiments, we examined the effect of LPA2 deficiency on T cell migration *in vivo* (see below).

### Lpa2-deficiency compromises T cell motility in HEV and in lymph nodes

**Figure 5:**
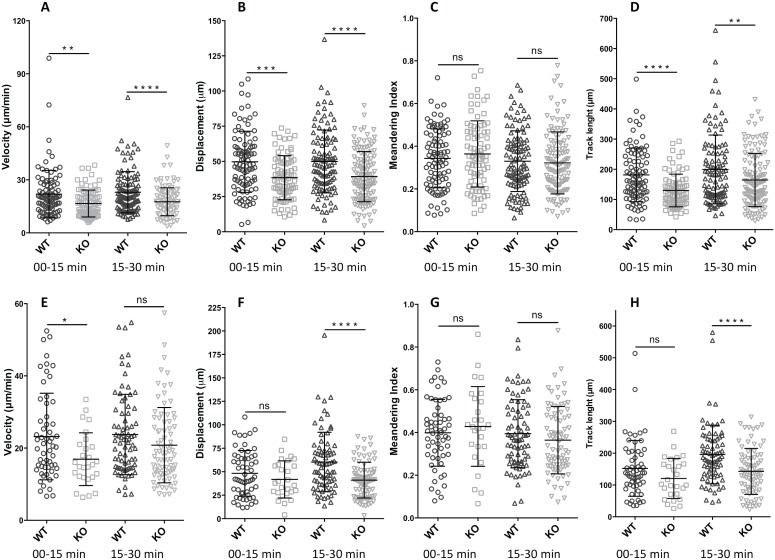
Lpa2-deficiency results in early defects in intranodal T cell migration. Naïve mouse CD4+ T cells from wild-type C57BL/6 mice and *lpa2−/−* mice were labeled with CFSE and CMTMR, respectively. (5–10)×10^6^ cells mixed at a 1∶1 ratio together with Texas Red Dextran were given to anesthetized wild-type recipients by injection into the orbital sinus immediately before imaging the microsurgically exposed popliteal lymph node. MP-IVM was performed to visualize intranodal T cell motility, and an HEV-specific mask was created. Images were acquired with a pixel dwell time of 2 µs, using step sizes of 2 µm to a depth of 50 µm every 45 seconds, and analyzed using Volocity software (see Methods). Data are the averaged±SEM of two independent experiments, represented separately for early (0–15 mins) and later (15–30 mins) phases of imaging of both intravascular (**A–D**) and intranodal (**E–H**) cells. *p<0.05, **p<0.005, ***p<0.0001.

The presence of LPA receptors on naïve CD4+ T cells and the observed ability of LPA to enhance T cell migration, together with the constitutive ATX production and presumably increased levels of LPA at the HEV, suggest a potential role for LPA-LPA receptor signaling during T cell homing *in vivo*. When T cells were incubated with enzymatically inactive ATX and subsequently injected into wild-type mice, short-term homing of the T cells to lymph nodes was attenuated (i.e. reduced distance migrated from HEV as assessed by histology) [2]. One possibility is that the inactive ATX could interfere with the function of endogenous ATX and exert a dominant negative effect, resulting in less local LPA production. However, it is unclear whether LPA acts on T cells, endothelial cells, or both at the HEV. In order to determine if LPA2-deficiency compromises naïve CD4+ T cell migration *in vivo*, we used intravital imaging with two-photon microscopy and compared WT with *lpa2*-deficient cells in competitive adoptive transfer. We labeled purified naïve CD4+ T cells from wild-type and *lpa2−/−* mice with CFSE and CMTMR fluorophores, respectively, mixed them in a 1∶1 ratio, and then injected them intravenously into wild-type recipient mice. Immediately after transferring the cells, we used two-photon intravital microscopy on exposed popliteal lymph nodes and imaged T cell behavior over 30 minutes (**Movie S1**). By monitoring depth of imaging and using Texas Red dextran to create an HEV-specific mask (see Methods), we separately analyzed intravascular T cells (**Figure 5A–D**) with extravascular, intranodal T cells (**Figure 5E–H**). Interestingly, compared to wild-type CD4+ T cells, intravascular *lpa2−/−* CD4+ T cells migrated significantly more slowly than their wild-type counterparts during the duration of imaging (**Figure 5A**). In addition, deficiency of LPA2 resulted in shorter overall displacement and track length, without significantly affecting meandering index (**Figure 5C**). Although the velocity of extravascular T cells was slower than intravascular T cells (as expected), intranodal motility of *lpa2−/−* CD4+ T cell was also significantly compromised compared to wild-type (**Figure 5E–H**). Taken together, these data suggest that LPA acting via *lpa2* plays has a previously unsuspected role in promoting intranodal CD4+ T cell motility both before and after extravasation from the vasculature.

### LPA2 deficiency does not compromise steady-state naïve CD4+ T cell homing to lymph nodes

**Figure 6:**
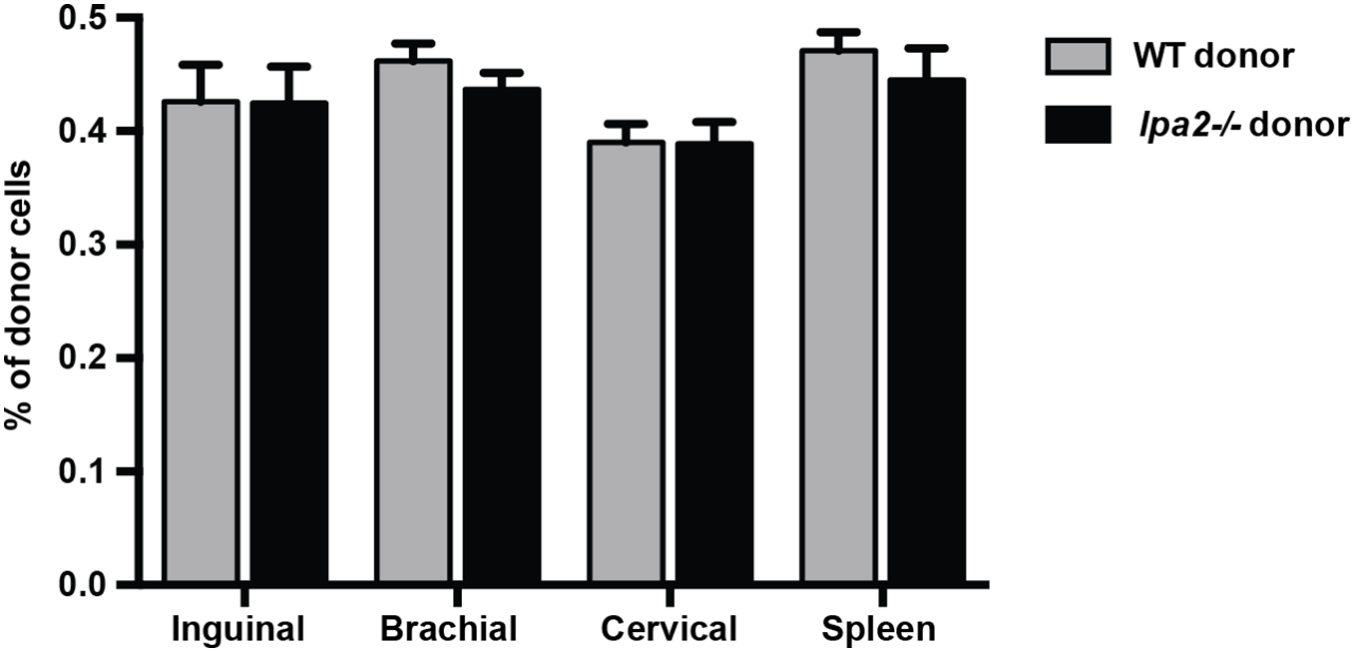
LPA2 does not play a crucial role in steady-state T cell homing to lymph nodes. Wild-type (CD90.2+, CD45.1+) and *lpa2*−/− (CD90.2+, CD45.1−) CD4+ T cells were adoptively transferred through tail vein injection into wild-type recipient mice. Forty-two hours post-transfer, the inguinal, brachial, cervical lymph nodes and spleen were harvested and the number of donor CD4+ T cells were enumerated by flow cytometry. Data are mean +/− SEM of two independent experiments, n = 5 recipient mice/experiment.

Deficiency of *lpa2* from birth does not affect the development of lymphocytes or secondary lymphoid organs [51]. This suggests that while there may be an acute defect in T cell migration and lymph node entry in the absence of *lpa2*, this might be overcome with time. To determine whether the absence of LPA2 affected steady-state CD4+ T cell recirculation, we performed a competitive adoptive transfer whereby wild-type naïve CD4+ T cells were co-transferred at a 1∶1 ratio with *lpa2−/−* naïve CD4+ T cells into recipient mice. Forty-two hours post-transfer, lymph nodes and spleens were collected and the frequency of donor cells in the recipient lymph nodes and spleen was determined by flow cytometry using unique congenic markers. Interestingly, there were no significant differences in the recovery of wild-type and *lpa2−/−* cells from the inguinal, brachial, axillary lymph nodes or spleen of recipient mice at these later time points (**Figure 6**).

## Discussion

Recent studies have implicated ATX and LPA in T cell migration at the HEV [2], [3], [33], [34]. One current model posits that ATX is produced and secreted by the HEV, producing a locally elevated concentration of LPA at the point of entry of lymphocytes. ATX has been shown to bind to the leading edge of human T cells in an integrin-dependent manner [2], as well as to the surface of endothelial cells [34]. If constitutive ATX expression by HEVs also generates high LPA levels due to the catalysis of endogenous LPC, then lymphocyte egress from the blood stream into the lymph node might be stimulated by engaging LPA receptors on circulating lymphocytes. Interestingly, the crystal structure of ATX was recently solved and revealed a hydrophobic channel extending from the active site where the alkyl chain of lipid substrates, such as LPA, can bind [52], [53]. While it is unclear how the newly formed LPA is specifically targeted to its cell surface receptors, this tunnel may serve as a product release site, with the ability to deliver LPA directly to its receptors within the HEV. It is currently not known if ATX-generated LPA acts on T cells, endothelial cells, or both, and different models have been proposed. For example, HEVs might create a “waiting area” where lymphocytes accumulate and are held before they eventually egress into the lymph node facilitated by LPA-induced changes in endothelial shape [54]. Based on data reported in this paper, we conclude that LPA can act directly on T cells to induce migration, and we identify previously unknown roles for LPA2 in this regard.

Goetzl et al. previously showed that CD4+ T cells isolated from healthy human donors express LPA2 but not LPA1 or LPA3, as determined by semi-quantitative PCR and Western blot [55]. LPA receptor expression and function on CD8+ T cells has not yet been widely studied. In a recent study Oda et al. found that mouse CD8+ T cells express LPA2, 5, and 6 and that LPA5 suppresses CD8+ T cell receptor activation, signaling, and tumor immunity [56]. The CD4+ T cell studies were performed before the identification of the P2Y family of receptors (LPA4–6) and the expression of all of the LPA receptors over the course of T cell activation has not been rigorously examined. LPA2 signaling has emerged as a potential feature in many different cancer pathways and is associated with promoting cell survival, proliferation, and angiogenesis (reviewed in [57]). LPA has also been shown to substitute for serum to markedly induce the invasion of T cell lymphoma cells through a fibroblast monolayer in a RhoA and PLC-dependent manner [58]. Transfecting LPA2 into Jurkat T cells enhanced LPA-induced chemotaxis and chemokinesis through a Matrigel, as a model of basement membrane [59]. LPA2 signals through multiple G proteins, and can stimulate cell motility via Gα12/13-dependent Rho activation and actin remodeling. Furthermore, LPA-induced activation of LPA2 can also lead to the recruitment of TRIP6, a focal adhesion molecule, to the C-terminus of LPA2 at the plasma membrane, leading to its phosphorylation and targeting to focal adhesions and co-localization with actin, thereby leading to cell adhesion and migration [60], [61]. However, when we compared wild-type and *lpa2*−/− CD4+ T cells in Transwell assays, we observed no difference in LPA-induced motility (**Figure 4**). It may be interesting in future experiments using chemoinvasion assays to determine if lpa2 facilitates T cell invasion through these solid matrices, and to contribute to our in vivo observation that *lpa2−/−* CD4+ T cells seem to get trapped at the border between the blood vessel and lymph node (discussed below).

Using Transwell assays we found that LPA does not induce chemotaxis of CD4+ T cells in vitro, but instead stimulates chemorepulsion (**Figure 1**). When LPA was added to both the top and the bottom chambers, directional migration of cells was abrogated suggesting that LPA was not inducing chemokinesis. These results differ from Kanda et al. who found that human T cells still migrated above baseline under similar conditions [2]. Since the number of replicates they reported was small [2], it is difficult to know whether this apparent discrepancy is due to species-specific differences in response to LPA or other factors. One potential confounding variable is the poor solubility of LPA in aqueous solutions, which can affect the bioavailability of LPA in tissue culture models. We used a carefully controlled approach (see Methods), and the results we report were consistent across multiple experiments and LPA lots.

Real-time imaging of lymphocyte migration at HEV and in lymph nodes has revolutionized our understanding of lymphocyte homing mechanisms [62]. Interestingly, when we visualized *lpa2*−/− CD4+ T cell migration at the HEV using two-photon intravital microscopy, we uncovered a previously unsuspected role for this receptor *in vivo*. The *lpa2*−/− T cells migrated at a significantly slower velocity and consistently traveled shorter distances during the time of imaging compared to WT CD4+T cells (Figure 5). This was true whether we compared intravascular T cells within the HEV as well as intranodal T cells within the node parenchyma. *lpa2*−/− CD4+ T cells seemed to get trapped at the border between the blood vessel and the lymph node compared to the wild-type T cells that extravasated through the HEV much more easily (Movie S1). These novel data suggest that *lpa2* plays an important role in promoting CD4+ T cell migration at the HEV at early time points after adoptive transfer. These findings likely help explain the previous observation that when T cells were incubated with an inactive mutant of ATX and adoptively transferred into recipient mice, these T cells homed less efficiently to the HEVs of lymph nodes at an early time point (15 minutes). Our results suggest that at least part of the effect of mutant ATX is explained by inhibiting local LPA production, which could act on LPA2 expressed by naïve CD4+ T cells traversing the HEV.

Although LPA2 appears to regulate T cell dynamics within lymph nodes at early stages after adoptive transfer, the fact that we recovered similar numbers of *lpa2*−/− and wild-type CD4+ T cells 42 hours after adoptive transfer (Figure 6) indicates that this receptor does not control steady-state T cell recirculation over time. We cannot exclude the possibility that deficiency of LPA2 compromises T cell localization or migratory behavior within lymph nodes at later stages after adoptive transfer, even if bulk recirculation patterns are unaffected. Future studies will be needed to determine if other LPA receptors compensate for the lack of *lpa2* over time, or if T cells were simply able to catch up over time independent of the influence of other receptors. What are the consequences of delayed migration of naïve CD4+ T cells within lymph nodes? The answer to this question will require further study, but the kinetics of T cell entry into secondary lymphoid organs could affect the quality or intensity of the effector response. In a naïve host, the kinetics of antigen entry into draining lymph nodes is variable and influenced by pathogen-specific features as well as degree of tissue infection or injury. For example, there is wide variation in timing of CD8+ T cell activation after different viral or bacterial infections: from 2 hours following HSV infection to within 10 days for TB infection [63]. After tissue damage, antigen can travel rapidly via bulk flow in afferent lymph and be taken up by sinusoidal macrophages, lymph node stromal cells, or conduit-associated DCs [64]–[66]. At later time points, cell-associated antigen enters lymph nodes transported by migrating tissue-derived APC’s [67]. Itano et al. demonstrated that early peptide:MHC II presentation by DCs that acquired antigen in the lymph node was sufficient to drive many aspects of T cell activation (first wave) prior to the arrival of antigen-loaded tissue resident DCs that activate a second wave of T cells [67]. Thus it seems likely that delaying the entry and/or migration of naïve T cell in lymph nodes will affect their encounter with antigen-bearing APC’s, with consequences for T cell activation.

Our discovery of a non-redundant role for LPA2 in T cell migration is important, since naïve CD4+ T cells express multiple LPA receptors. Future studies using gene-targeted mice and specific receptor inhibitors will help to dissect the individual roles of each LPA receptor on CD4+ T cell immune responses. There is likely cross-talk between the different LPA receptors in a cell-type specific manner, as well as interactions with other G-protein coupled receptors that regulate T cell migration [68]. Our results add to the growing body of literature documenting an important role for LPA in the immune system, and suggest that future studies of LPA generation and action *in vivo* will enhance our understanding of initiation of immune responses.

## Supporting Information

Movie S1 **Lpa2-deficiency results in early defects in T cell migration at the HEV (Supplemental to** **Figure 5** **).** Representative two-photon microscopic images obtained from the popliteal lymph node of wild-type mice. Wild-type and lpa2−/− CD4+ T cells were labeled with CFSE and CMTMR, respectively and (5–10)×10^6^ cells mixed in a 1∶1 ratio together with Texas Red Dextran were injected into wild-type recipients through the orbital sinus. MP-IVM was performed on a microsurgically exposed popliteal lymph node to visualize T cell motility during extravasation at the HEV. The video shows the first 31 minutes of imaging, which is representative of migration over the course of an hour.
(MOV)
